# Estimating neural response functions from fMRI

**DOI:** 10.3389/fninf.2014.00048

**Published:** 2014-05-08

**Authors:** Sukhbinder Kumar, William Penny

**Affiliations:** ^1^Wellcome Trust Centre for Neuroimaging, University College LondonLondon, UK; ^2^Medical School, Institute of Neuroscience, Newcastle UniversityNewcastle, UK

**Keywords:** neural response function, population receptive field, parametric modulation, Bayesian inference, auditory perception, repetition suppression, Tonotopic Mapping, Balloon model

## Abstract

This paper proposes a methodology for estimating Neural Response Functions (NRFs) from fMRI data. These NRFs describe non-linear relationships between experimental stimuli and neuronal population responses. The method is based on a two-stage model comprising an NRF and a Hemodynamic Response Function (HRF) that are simultaneously fitted to fMRI data using a Bayesian optimization algorithm. This algorithm also produces a model evidence score, providing a formal model comparison method for evaluating alternative NRFs. The HRF is characterized using previously established “Balloon” and BOLD signal models. We illustrate the method with two example applications based on fMRI studies of the auditory system. In the first, we estimate the time constants of repetition suppression and facilitation, and in the second we estimate the parameters of population receptive fields in a tonotopic mapping study.

## 1. Introduction

Functional Magnetic Resonance Imaging (fMRI) is a well established technique for the non-invasive mapping of human brain function (Frackowiak et al., 2003). Analysis of fMRI data most often proceeds by modeling the neuronal correlates of single events as delta functions or boxcars. These form event streams which are then convolved with assumed Hemodynamic Response Functions (HRFs) to create regressors for General Linear Models (GLMs). This forms the basis of the widely used Statistical Parametric Mapping (SPM) approach (Friston et al., 2007).

**Figure 1:**
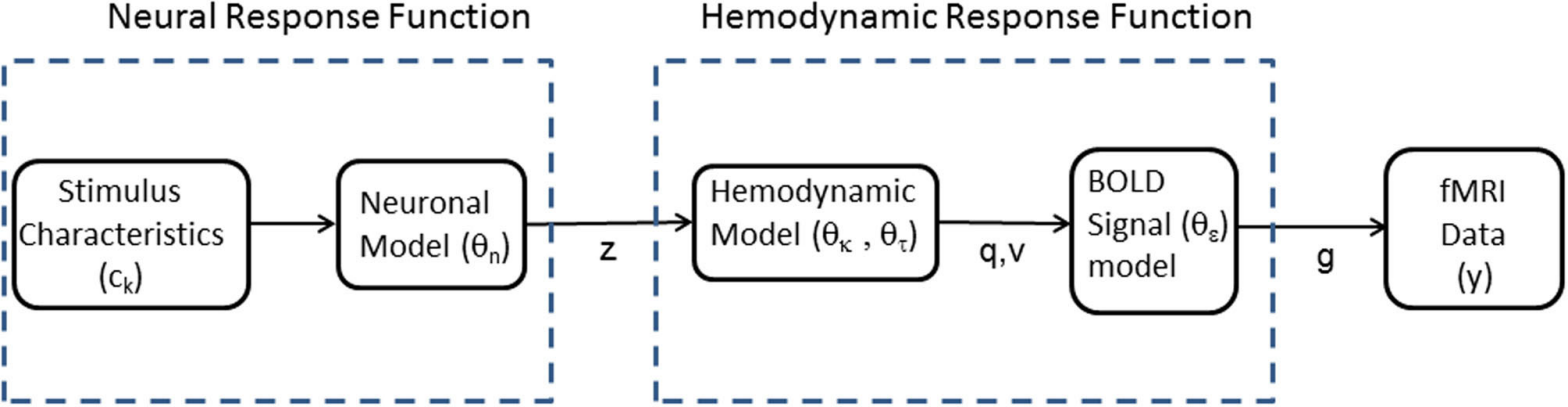
**Modular structure of generative model**. Our framework proposes voxel-wise or region-wise fitting of a non-linear model to fMRI data. The model comprises separate modules which characterize a Neural Response Function (NRF) and a Hemodynamic Response Function (HRF). The NRF is based on a non-linear parametric form, which will vary depending on the application domain, relating neuronal activity *z* to known stimulus characteristics, *c_k_*, and unknown neuronal parameters θ_*n*_. The HRF relates predicted fMRI activity *g* to neuronal activity *z* via blood deoxyhemoglobin *q* and volume *v* variables. These relations are specified by an extended Balloon model with unknown parameters θ_*h*_ = {θ_κ_, θ_τ_, θ_ϵ_} describing the rate of signal decay, transit time, and ratio of intra- to extra-vascular signal, respectively. The model is fitted to fMRI data, *y*, so as to estimate the unknown parameters θ_*n*_ and θ_*h*_.

This paper proposes an alternative approach in which fMRI data is fitted using a non-linear model depicted in Figure 1. This comprises two mappings (1) a Neural Response Function (NRF) which maps stimulus characteristics to neural responses and (2) an HRF which maps neural responses to fMRI data. Importantly, the HRF can accommodate variations across brain regions and subjects, and includes non-linearities associated with hemodynamic saturation effects. The parameters of the two mappings are estimated together using a Bayesian optimization algorithm that is widely used in neuroimaging (Friston et al., 2007). This algorithm has the added benefit of producing a model evidence score which we will use to provide a formal model comparison method (Penny, 2012) for evaluating alternative NRFs.

The goal of our approach is to make inferences about NRFs. These parametric models relate the activity of a population of neurons within a single voxel or brain region to characteristics of experimental stimuli. NRFs are similar, in principle, to those derived for individual neurons from single unit electrophysiology but estimate population rather than single neuron responses (Dumoulin and Wandell, 2008). In this paper we apply the NRF approach to the auditory domain and provide two examples. The first is a Repetition Suppression paradigm in which we estimate neural responses as a function of time since presentation of a similar stimulus. These repetition suppression effects are an important marker of synaptic plasticity (Weigelt et al., 2008; Marta et al., 2009). The second is a Tonotopic Mapping paradigm in which we model neural responses as Gaussian or Mexican-Hat functions of stimulus frequency, and report the results of a formal Bayesian model comparison.

This paper is based on a previous Hemodynamic Model (HDM) (Friston, 2002), which posited categorical relations between stimuli and neural activation, and used a biophysically motivated differential equation model of the HRF, which in turn was based on earlier physiological modeling (Buxton et al., 1998). This paper can be viewed as a simple extension of that work which replaces the categorical neuronal model with a parametric one.

A further perspective on this paper is that it presents an extension of linear models with “Parametric Modulation” terms, in which experimental variables of interest are used to modulate the height or duration of boxcar functions representing neuronal activity (Buchel et al., 1998; Grinband et al., 2008). The work in this paper represents an extension of this approach by allowing for non-linear relations between fMRI signals and unknown parametric variables. Non-linear relationships can also be accommodated in the linear framework by using a Taylor series approach, but this has a number of disadvantages which are described in section 2.

## 2. Materials and methods

Figure 1 shows the structure of the model proposed in this paper. An NRF specifies how neuronal activity is related to stimulus characteristics and an HRF specifies how fMRI data is related to neuronal activity. The HRF is based on the Balloon model (Buxton et al., 1998) which describes how blood deoxyhemoglobin, *q*, and volume, *v*, are driven by neuronal activity, and a BOLD signal model which describes how the BOLD signal derives from *q* and *v*. The hemodynamic and BOLD signal models are the same as those used in Dynamic Causal Modeling (DCM) (Stephan et al., 2007).

The sub-sections below describe the above modules in more detail. We also briefly describe the optimization algorithm used to fit the model to fMRI data. This is a Bayesian estimation procedure which also requires the specification of prior distributions over model parameters. We also provide a description of the Taylor series approach for estimation of non-linear parametric functions. In what follows N(*x; m, S*) denotes a multivariate Gaussian distribution over variable *x* with mean *m* and covariance *S*, and U(*x; l, u*) denotes a univariate uniform density with lower and upper bounds *l* and *u*.

### 2.1. Neural response functions

The original formulation of the Hemodynamic Model (HDM) (Friston, 2002) considered categorical relationships between experimental manipulations and neuronal activity. For the *k*th experimental condition the neuronal time series is modeled as

(1) $$z_{k}(t)=\beta_{k}\sum_{j=1}^{N_{k}}\delta [t-t_{k}(j)]$$

where *t_k_(j)* are the event times for condition *k*, *N_k_* is the number of condition *k* events, and δ[] is a delta function. The variable *t* denotes time in seconds since the beginning of the fMRI recording session, and event times *t_k_(j)* are specified in the same units. The parameter β_*k*_ is the “neuronal efficacy” for condition *k* and indicates the magnitude of the neural response.

This paper extends the HDM formalism by also allowing for non-linear parametric relationships. For example, for the Repetition Suppression paradigm we use a model of the form

(2) $$z_{k}(t)=\beta_{k}\sum_{j=1}^{N_{k}}\delta [t-t_{k}(j)]\exp[-a_{k}r_{k}(j)]$$

with a parametric variable *r_k_(j)* denoting the time-lag (seconds) or item-lag (number of repeats) associated with the *j*th event in condition *k*. The variable *a_k_* is the associated time constant that we wish to estimate from data, with positive values indicating suppression and negative values facilitation. The total neuronal activity is then given by

(3) $$z(t)=\sum_{k}z_{k}(t)+\beta_{0}$$

where β_0_ is baseline neural activity. The incorporation of this baseline parameter is also novel to this paper and we show in section 3 that it can significantly improve model fit. Overall neuronal activity, and the resulting BOLD signal (see below), are non-linear functions of the parameter *a_k_*. This is therefore an example of a non-linear parametric response.

For our repetition suppression experiment (see section 2.8) there are *k* = 1..4 experimental conditions denoting the four different types of auditory stimulus. There are multiple trials, *N_k_*, for each condition. In our experiment the different conditions are presented in “pitch trains.” As the stimuli in the different trains are presented several seconds apart, the linear summation in Equation (3) merely combines the condition specific neural responses, *z_k_*, into a single variable, *z*. Had the stimuli for the different categories been interleaved in close temporal proximity this assumption would be questionable (Friston et al., 1998).

For the Tonotopic Mapping paradigm we model neural responses as a Gaussian function of stimulus frequency

(4) $$z(t)=\beta \sum_{j=1}^{N}\delta [t-t(j)]\exp\left[-\frac{1}{2}{\left(\frac{f_{j}-\mu }{\sigma }\right)}^{2}\right]+\beta_{0}$$

where *f_j_* is the stimulus frequency (in Hz) for the *j*th event, μ is the center frequency (of the population receptive field), σ is the width and β is the magnitude. We also explore a Mexican-Hat wavelet parametric form

(5) $$\begin{array}{l} z(t)=\beta \sum_{j=1}^{N}\delta [t-t(j)]\left[1-{\left(\frac{f_{j}-\mu }{\sigma }\right)}^{2}\right]\\ \;\exp\left[-\frac{1}{2}{\left(\frac{f_{j}-\mu }{\sigma }\right)}^{2}\right]+\beta_{0} \end{array}$$

This function, also referred to as a Ricker wavelet, is equivalent to the (negative, normalized) second derivative of a Gaussian function (Mallat, 1999). The function corresponds to a Gaussian with surround suppression and can also be produced using a Difference of Gaussians (DoG) functional form (with specific parameter settings). Population receptive fields with surround suppression have been explored in the visual domain (Lee et al., 2013). Overall neuronal activity, and the resulting BOLD signal (see below), are non-linear functions of the parameters μ and σ. This is therefore another example of a non-linear parametric response. For the Tonotopic Mapping data we treat all stimuli as belonging to the same category, so have dropped the *k* subscripts in Equations (4, 5).

Although this paper focusses on the auditory system, we envisage that our approach may also be useful for many other types of neuroimaging study. So, generally we allow for NRFs of the form

(6) $$z(t)=f\left(c_{1},c_{2},\ldots ,c_{K};\theta_{n}\right)$$

where *c_k_* are stimulus characteristics for conditions *k* = 1…*K* and *f* is an arbitrary linear or non-linear function with parameters θ_*n*_. For our Repetition Suppression example the neuronal parameters are θ_*n*_ = {*a*_*k*_, β_*k*_, β_0_} and for the Tonotopic Mapping example they are θ_*n*_ = {μ, σ, β, β_0_} (here μ, σ and β are specified indirectly by Gaussian latent variables to allow for an appropriately constrained optimization, as described in section 2.3 below). More generally, the functional form is to be provided by the modeler and the parameters are to be estimated, as described below. Different NRFs (e.g., Gaussian versus Mexican-Hat) can then be evaluated in relation to each other using Bayesian Model Comparison (Penny, 2012).

### 2.2. Hemodynamics

Neuronal activity gives rise to fMRI data by a dynamic process described by an extended Balloon model (Buxton et al., 2004) and BOLD signal model (Stephan et al., 2007) for each brain region. This specifies how changes in neuronal activity give rise to changes in blood oxygenation that are measured with fMRI.

The hemodynamic model involves a set of hemodynamic state variables, state equations and hemodynamic parameters, θ_*h*_. Neuronal activity *z* causes an increase in vasodilatory signal *s* that is subject to autoregulatory feedback and inflow *f*_*in*_ responds in proportion to this

(7) $$\begin{array}{l} \;\dot{s}=z-\kappa s-\gamma (f_{in}-1)\\ {\dot{f}}_{in}=s \end{array}$$

Blood volume *v* and deoxyhemoglobin content *q* then change according to the Balloon model

(8) $$\begin{array}{l} \tau \dot{v}=f_{in}-f_{out}\\ \tau \dot{q}=f_{in}E(f_{in},\rho )-f_{out}\frac{q}{v} \end{array}$$

(9) $$f_{out}=v^{1/\alpha }$$

where the first equation describes the filling of the venous “Balloon” until inflow equals outflow, *f*_*out*_, which happens with time constant τ. The proportion of oxygen extracted from the blood is a function of flow

(10) $$E(f,\rho )=\frac{1-{\left(1-\rho \right)}^{1/f}}{\rho }$$

where ρ is resting oxygen extraction fraction. The free parameters of the model are the rate of signal decay in each region, κ, and the transit time in each region, τ. The other parameters are fixed to γ = α = ρ = 0.32 in accordance with previous work (Stephan et al., 2007).

#### 2.2.1. BOLD signal model

The BOLD signal is given by a static non-linear function of volume and deoxyhemoglobin that comprises a volume-weighted sum of extra- and intra-vascular signals. This is based on a simplified approach (Stephan et al., 2007) (Equation 12) that improves upon an earlier model (Friston et al., 2003)

(11) $$\begin{array}{l} y=V_{0}\left[k_{1}(1-q)+k_{2}\left(1-\frac{q}{v}\right)+k_{3}(1-v)\right]\\ k_{1}=4.3\theta_{0}\rho TE\\ k_{2}=\epsilon r_{0}\rho TE\\ k_{3}=1-\epsilon \end{array}$$

where *V*_0_ is resting blood volume fraction, θ_0_ is the frequency offset at the outer surface of the magnetized vessel for fully deoxygenated blood at 1.5T, TE is the echo time and *r*_0_ is the slope of the relation between the intravascular relaxation rate and oxygen saturation (Stephan et al., 2007). In this paper we use the standard parameter values *V*_0_ = 4, *r*_0_ = 25, θ_0_ = 40.3 and for our fMRI imaging sequence we have *TE* = 0.04. The only free parameter of the BOLD signal model is ϵ, the ratio of intra- to extra-vascular signal.

### 2.3. Priors

The overall model is fitted to data using the Variational Laplace (VL) optimization algorithm (Friston et al., 2007). This is a Bayesian estimation procedure which requires the specification of prior distributions over model parameters. The algorithm is widely used in neuroimaging, finding applications ranging from fitting of Equivalent Current Dipole source models to DCMs (Litvak et al., 2011). Within VL, priors must be specified as Gaussians (see section 2.5). However, priors of any unimodal form can in effect be specified over variables of interest by using Gaussian latent variables and the appropriate non-linear transform. For example, we use uniform priors over parameters of the Tonotopic models (see below).

#### 2.3.1. Neural response function

In the absence of other prior information about NRF parameters we can initially use Gaussian priors with large variances, or uniform priors over a large range. Applying the optimization algorithm to selected empirical fMRI time series then provides us with ballpark estimates of parameter magnitudes. The priors can then be set to reflect this experience (Gelman et al., 1995). Alternatively, one may be able to base these values on published data from previous studies.

For the Repetition Suppression models used in this paper, we use the following priors. The initial effect size has a Gaussian prior

(12) $$p(\beta_{k})=\text{N}\left(\beta_{k};1,\sigma_{\beta }^{2}\right)$$

with σ^2^_β_ = 10, and the decay coefficient also has a Gaussian prior

(13) $$p(a_{k})=\text{N}\left(a_{k};0,\sigma_{a}^{2}\right)$$

with σ^2^_*a*_ = 1. The baseline neuronal activity also has a Gaussian prior

(14) $$p(\beta_{0})=\text{N}\left(\beta_{0};0,\sigma_{\beta }^{2}\right)$$

For the Tonotopic Mapping examples we used uniform priors over the center frequency, width, and amplitude as follows

(15) $$\begin{array}{l} p(\mu )=\text{U}(\mu ;\mu_{min},\mu_{max})\\ p(\sigma )=\text{U}(\sigma ;\sigma_{min},\sigma_{max})\\ p(\beta )=\text{U}(\beta ;\beta_{min},\beta_{max}) \end{array}$$

The minimum and maximum values were μ_*min*_ = 0, μ_*max*_ = 20, 000, σ_*min*_ = 1, σ_*max*_ = 5, 000, β_*min*_ = 0, β_*max*_ = 20. The center frequency and width are expressed in Hz. These uniform priors were instantiated in the VL framework by specifying a Gaussian latent variable and relating model parameters to latent variables via the required non-linearity. We used

(16) $$\begin{array}{l} \mu =(\mu_{max}-\mu_{min})\Phi (\theta_{\mu })+\mu_{min}\\ \sigma =(\sigma_{max}-\sigma_{min})\Phi (\theta_{\sigma })+\sigma_{min}\\ \beta =(\beta_{max}-\beta_{min})\Phi (\theta_{\beta })+\beta_{min} \end{array}$$

The priors over the latent variables θ_μ_, θ_σ_, and θ_β_ were standard Gaussians (zero mean, unit variance). The required non-linearity Φ was therefore set to the standard cumulative Gaussian function (Wackerley et al., 1996). The prior over β_0_ was given by Equation (14).

In summary, for the Repetition Suppression example the neuronal parameters are θ_*n*_ = {*a*_*k*_, β_*k*_, β_0_} and for the Tonotopic Mapping example they are θ_*n*_ = {θ_μ_, θ_σ_, θ_β_, β_0_}.

#### 2.3.2. Hemodynamic response function

The unknown parameters are {κ, τ, ϵ}. These are represented as

(17) $$\begin{array}{l} \kappa =0.64\exp\text{​}(\theta_{\kappa })\\ \tau =2\exp\text{​}(\theta_{\tau }) \end{array}$$

$$\epsilon =\exp\text{​}(\theta_{\epsilon })$$

and we have Gaussian priors

(18) $$\begin{array}{l} p(\theta_{\kappa })=\text{N}(\theta_{\kappa };0,0.135)\\ p(\theta_{\tau })=\text{N}(\theta_{\tau };0,0.135)\\ p(\theta_{\epsilon })=\text{N}(\theta_{\epsilon };0,0.135) \end{array}$$

where θ_*h*_ = {θ_κ_, θ_τ_, θ_ϵ_} are the hemodynamic parameters to be estimated. These priors are used for both the applications in this paper and are identical to those used in DCM for fMRI.

### 2.4. Integration

Our overall parameter vector θ = {θ_*n*_, θ_*h*_} comprises neurodynamic and hemodynamic parameters. Numerical integration of the hemodynamic equations leads to a prediction of fMRI activity comprising a single model prediction vector *g*(θ, *m*). This has dimension [*T* × 1] where *T* is the number of fMRI scans (length of time series). The numerical integration scheme used in this paper is the ode15s stiff integrator from Matlab's ODE suite (Shampine and Reichelt, 1997).

### 2.5. Optimization

The VL algorithm can be used for Bayesian estimation of non-linear models of the form

(19) y=g(θ,m)+e

where *y* is the fMRI time series, *g*(θ, *m*) is a non-linear function with parameters θ, and *m* indexes assumptions about the NRF. For example, in the Repetition Suppression example below (see section 3) *m* indexes “item-lag” or “time-lag” models, and in the Tonotopic Mapping example *m* indexes Gaussian or Mexican-Hat parametric forms.

The term *e* denotes zero mean additive Gaussian noise. The likelihood of the data is

(20) $$p(y|\theta ,\lambda ,m)=\text{N}(y;g(\theta ,m),\exp\text{​}{\left(\lambda \right)}^{-1}I_{T})$$

with noise precision exp(λ) and *p*(λ|*m*) = N(λ; μ_λ_, *S*_λ_) with μ_λ_ = 0, *S*_λ_ = 1. Here *I_T_* denotes a dimension *T* identity matrix. These values are used in DCM (Penny, 2012) and have been set so as to produce data sets with signal to noise ratios that are typical in fMRI.

The framework allows for Gaussian priors over model parameters

(21) $$p(\theta |m)=\text{N}(\theta ;\mu_{\theta },C_{\theta })$$

where μ_θ_ and *C*_θ_ have been set as described in the previous section on priors.

These distributions allow one to write down an expression for the joint log likelihood of data, parameters and hyperparameters

(22) p(y,θ,λ|m)=p(y|θ,λ,m)p(θ|m)p(λ|m)

The VL algorithm then assumes an approximate posterior density of the following factorized form

(23) $$\begin{array}{l} q(\theta ,\lambda |y,m)=q(\theta |y,m)q(\lambda |y,m)\\ \;q(\theta |y,m)=\text{N}(\theta ;m_{\theta },S_{\theta })\\ \;q(\lambda |y,m)=\text{N}(\lambda ;m_{\lambda },S_{\lambda }) \end{array}$$

The parameters of these approximate posteriors are then iteratively updated so as to minimize the Kullback-Leibler (KL)-divergence between the true and approximate posteriors. This is the basic principle underlying all variational approaches to approximate Bayesian inference; that one chooses a factorization of the posterior and updates parameters of the factors (here *m*_θ_, *S*_θ_, *m*_λ_, and *S*_λ_) so as to minimize the KL-divergence. Readers unfamiliar with this general approach can find introductory material in recent texts (Jaakola et al., 1998; Bishop, 2006). For the VL algorithm, this minimization is implemented by maximizing the following “variational energies”

(24) $$\begin{array}{l} I(\theta )=\int L(\theta ,\lambda )q(\lambda |y,m)d\lambda \\ I(\lambda )=\int L(\theta ,\lambda )q(\theta |y,m)d\theta \end{array}$$

where *L*(θ, λ) = log*p*(*y*, θ, λ|*m*). As the likelihood, priors, and approximate posteriors are Gaussian the above integrals can be computed analytically (Bishop, 2006). Maximization is effected by first computing the gradient and curvature of the variational energies at the current parameter estimate, *m*_θ_(*old*). For example, for the parameters we have

(25) $$\begin{array}{l} \;j_{\theta }(i)=\frac{\partial I(\theta )}{\partial \theta (i)}\\ H_{\theta }(i,j)=\frac{d^{2}I(\theta )}{\partial \theta (i)\partial \theta (j)} \end{array}$$

where *i* and *j* index the *i*th and *j*th parameters, *j*_θ_ is the gradient vector and *H*_θ_ is the curvature matrix. These gradients and curvatures are computed using central differences (Press et al., 1992). In recent work (Sengupta et al., in press) we have proposed a more efficient “adjoint method,” which computes gradients and curvatures as part of the numerical integration process.

The estimate for the posterior mean is then given by

(26) $$m_{\theta }(new)=m_{\theta }(old)-H_{\theta }^{-1}j_{\theta }$$

which is equivalent to a Newton update (Press et al., 1992). In regions of parameter space near maxima the curvature is negative definite (hence the negative sign above). Equation (26) implements a step in the direction of the gradient with a step size given by the inverse curvature. Large steps are therefore taken in regions where the gradient changes slowly (low curvature) and small steps where it changes quickly (high curvature). In the SPM implementation (in the function spm_nlsi_GN.m from http://www.fil.ion.ucl.ac.uk/spm/), the update also incorporates a regularization term (Press et al., 1992). Readers requiring a complete description of this algorithm are referred to Friston et al. (2007).

A key feature of our approach, in which neurodynamic and hemodynamic parameters are estimated together rather than in a sequential “two-step” approach, can be illustrated by a closer inspection of Equation (26). If we decompose the means, gradients and curvatures into neurodynamic and hemodynamic parts

(27) $$\begin{array}{l} m_{\theta }={\left[m_{n},m_{h}\right]}^{T}\\ \;j_{\theta }={\left[j_{n},j_{h}\right]}^{T}\\ H_{\theta }=[H_{nn},H_{nh};H_{hn},H_{hh}] \end{array}$$

then [using the Schur complement (Bishop, 2006)] we can write the update for the neurodynamic parameters as

(28) $$m_{n}(new)=m_{n}(old)-{\left[H_{nn}-H_{nh}H_{hh}^{-1}H_{hn}\right]}^{-1}j_{n}$$

whereas the equivalent second step of a two-step approach would use

(29) $$m_{n}(new)=m_{n}(old)-H_{nn}^{-1}j_{n}$$

Thus, the joint estimation procedure includes an additional term such that components of the data that are explained by hemodynamic variation are not attributed to a neuronal cause. If there is no correlation between hemodynamic and neuronal parameters then *H*_*nh*_ = *H_hn_* = 0 and this additional term disappears. This is similar to the issue of how correlated predictors are dealt with in General Linear Modeling (Christensen, 2002; Friston et al., 2007).

### 2.6. Model comparison

The VL algorithm also computes the model evidence *p*(*y|m*) based on a free-energy approximation (Penny, 2012). Given models *m* = *i* and *m* = *j* the Bayes factor for *i* versus *j* is then given by Kass and Raftery (1995)

(30) $$BF_{ij}=\frac{p(y|m=i)}{p(y|m=j)}$$

When *BF*_*ij*_ > 1, the data favor model *i* over *j*, and when *BF*_*ij*_ < 1 the data favor model *j*. If there are more than two models to compare then we can choose one as a reference and calculate Bayes factors relative to that reference. A Bayes factor greater than 20 or, equivalently, a log Bayes factor greater than 3 is deemed strong evidence in favor of a model (Raftery, 1995). It is also possible to compute Bayes factors using the Savage–Dickey method, which only requires fitting a single “grandfather” model (Penny and Ridgway, 2013). The use of Bayes factors provides a Bayesian alternative to hypothesis testing using classical inference (Dienes, 2011).

### 2.7. Taylor series approximation

Previously in the neuroimaging literature a Taylor series approximation method has been used to estimate parameters that relate stimulus properties non-linearly to neuronal activity (Buchel et al., 1998). One can apply this approach, for example, to estimating time constants in Repetition Suppression experiments. For example, if we take Equation (2) (but drop reference to condition *k* for brevity) we have

(31) $$z(t)=\beta \sum_{j=1}^{N}\delta [t-t(j)]\exp[-ar(j)]$$

A first order Taylor series expansion (in the variable *a*) of the exponential function around an assumed value *a*_0_ then gives

(32) $$\begin{array}{l} z(t)\approx \beta \sum_{j=1}^{N}\delta [t-t(j)](\exp[-a_{0}r(j)]\\ \;-(a-a_{0})r(j)\exp[-a_{0}r(j)]) \end{array}$$

This can be written as

(33) $$\begin{array}{l} \;z(t)=\beta_{1}z_{1}(t)+\beta_{2}z_{2}(t)\\ \;\beta_{1}=\beta \\ \;\beta_{2}=\beta (a-a_{0})\\ z_{1}(t)=\sum_{j=1}^{N}\delta [t-t(j)]\exp[-a_{0}r(j)]\\ z_{2}(t)=-\sum_{j=1}^{N}\delta [t-t(j)]r(j)\exp[-a_{0}r(j)] \end{array}$$

Convolution of this activity then produces the predicted BOLD signal

(34) $$\begin{array}{l} g(t)=\beta_{1}x_{1}(t)+\beta_{2}x_{2}(t)\\ \;x_{1}=z_{1}⊗h\\ \;x_{2}=z_{2}⊗h \end{array}$$

where *h* is the hemodynamic response function (assumed known). This also assumes linear superposition (that the response of a sum is the sum of responses). This linearized model can be fitted to fMRI data using a standard GLM framework, with design matrix columns *x*_1_ and *x*_2_ and estimated regression coefficients ${\hat{\beta }}_{1},\;{\hat{\beta }}_{2}$. The estimated time constant is then given by

(35) $$\hat{a}=\frac{{\hat{\beta }}_{2}}{{\hat{\beta }}_{1}}+a_{0}$$

The drawbacks of this approach are (1) it assumes that a reasonably accurate estimate of *a* can be provided (*a*_0_, otherwise the Taylor series approximation is invalid), (2) it assumes the hemodynamic response is known and fixed across voxels, (3) it assumes linear superposition (e.g., neglecting possible hemodynamic saturation effects), and (4) inference is not straightforward as the parameter estimate is based on a ratio of estimated quantities. However, the great benefit of this approach is that estimation can take place using the GLM framework, allowing efficient application to large areas of the brain.

### 2.8. Repetition suppression data

The experimental stimuli consisted of three pitch evoking stimuli with different “timbres;” Regular Interval Noise (RIN), harmonic complex (HC), and regular click train (CT). Five different pitch values were used having fundamental frequencies equally spaced on a log-scale from 100 to 300 Hz. The duration of each stimulus was 1.5 s.

The RIN of a pitch value F0 was generated by first generating a sample of white noise, delaying it by 1/F0 s and then adding it back to the original sample. This delay and add procedure was repeated 16 times to generate a salient pitch. The stimulus was then bandpass filtered to limit its bandwidth between 1000 and 4000 Hz. New exemplars of white noise were used to generate RIN stimuli that were repeated within trials.

The HC stimulus of fundamental frequency F0 was generated by adding sinusoids of harmonic frequencies (multiples of F0) up to a maximum frequency (half the sampling rate) with phases chosen randomly from a uniform distribution. The resulting signal was then Bandpass filtered between 1000 and 4000 Hz.

The CT of rate F0 consisted of uniformly spaced bursts of clicks (click duration 0.1 ms) with interval duration (time between clicks) equal to 1/F0 s. This train of clicks was then bandpass filtered between 1000 and 4000 Hz.

We also included spectrally matched white noise stimuli (Noise) which were bandlimited to the same frequency range as pitch stimuli. Different white noise exemplars were used to generate each RIN and Noise stimulus.

**Figure 2:**
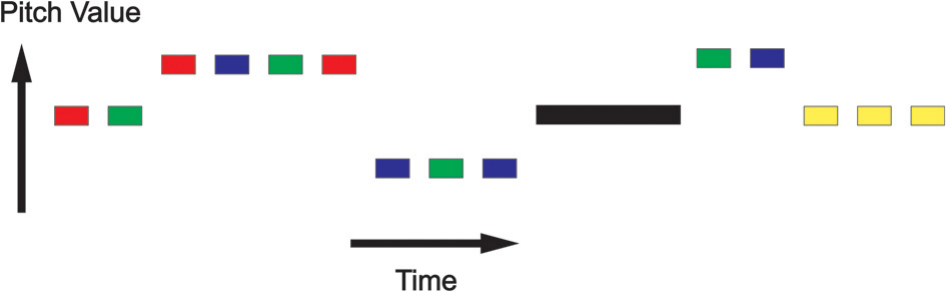
**Repetition suppression paradigm**. Stimuli were presented in epochs with periods of silence in between. Within each epoch, stimuli were presented in “pitch-trains” or “noise-trains,” where a pitch-train contained between 1 and 6 stimuli of the same pitch, and noise-trains contained between 1 and 6 noise stimuli. Colors indicate the various types of pitch train: Random Interval Noise (RIN) (red), Harmonic Complex (HC) (green), Click Train (CT) (blue), and noise (yellow). The black rectangle represents a period of silence.

Stimuli were presented in epochs with periods of silence in between, as shown in Figure 2. Within each epoch, stimuli were presented in “pitch-trains” or “noise-trains,” where a pitch-train contained between 1 and 6 stimuli of the same pitch, and noise-trains contained between 1 and 6 noise stimuli. For the HC and RIN stimuli, although the pitch value remained the same in each pitch-train, the low level acoustic structure varied over stimuli. For the CT stimuli, however, the low level structures were identical.

All imaging data were collected on a Siemens 3 Tesla Allegra head-only MRI scanner. The participant gave written consent and the procedures were approved by the University College London Ethics committee. Stimuli were presented as shown in Figure 2, with MRI data being continuously acquired from 30 slices covering the superior temporal plane (TR = 1.8 s, TE = 30 ms, FA = 90°, isotropic voxel size = 3 mm). To ensure subjects attended to the stimuli, they were asked to press a button at the start of each silent period. The scanning time was divided into 5 sessions, each lasting about 12 min. A total of 1800 volumes were acquired (360 per session).

After discarding the first 2 dummy images to allow for T1 relaxation effects, images were realigned to the first volume. The realigned images were normalized to stereotactic space and smoothed by an isotropic Gaussian kernel of 6 mm full-width at half maximum.

Cytoarchitectonically, Heschl's gyrus (HG) can be partitioned into three different areas (Morosan et al., 2001): a primary area (TE10) surrounded by two medial (TE11) and lateral areas (TE12) (see Figure 11 in Morosan et al., 2001). To test whether these three areas have different rates of adaptation to the repetition of pitch and noise stimuli, we extracted a time series from each of these areas. The anatomical mask of these areas, available in the anatomy toolbox (Eickhoff et al., 2005), were used to define the ROIs. Principal component analysis was carried out to summarize multiple time series (from multiple voxels in a ROI) to a single time series by the first principal component.

It is well known that repeated presentation of a stimulus leads to adaptation of brain responses (Buckner et al., 1998; Grill-Spector et al., 1999). These neural adaptation or repetition suppression (RS) effects are described in a recent review (Weigelt et al., 2008). In this paper we tested for Repetition Suppression effects by estimating exponential neural response functions, as described in section 3. We modeled neural activity as a function of repetition number (“item-lag”) or repetition time (“time-lag”) within each epoch, and our aim was to estimate the associated time constant. Although stimuli varied in pitch this was neglected for the current analysis.

### 2.9. Tonotopic mapping data

The stimuli for the tonotopic mapping consisted of 14 pure tones of frequencies: 88, 125, 177, 250, 354, 500, 707, 1000, 1414, 2000, 2828, 4000, 5657, and 8000 Hz. Starting from a frequency of 88 Hz, bursts of each tone were presented for 2 s after which the frequency was increased to the next highest frequency until all 14 frequencies were presented in a single block of 28 s. The block of sound was followed by a 12 s silent period. This cycle of 40 s was repeated 15 times in a single session lasting 10 min. Stimuli were presented using sensimetrics earphones (http://www.sens.com/products/model-s14/) at a sampling rate of 44,100 Hz.

Imaging data were acquired on Siemens 3 Tesla Quattro head-only MRI scanner. The MRI images were acquired continuously using 3D MRI sequence (TR = 1.1 s, two echoes per image; TE1 = 15.85 ms; TE2 = 34.39 ms; matrix size = 64 × 64). A total of 560 volumes were acquired in one session. After the fMRI run, a high resolution (1 × 1 × 1 mm) T1-weighted structural MRI scan was acquired. The two echoes of the images were first averaged. The images were then pre-processed in the same way as the Repetition Suppression data. We restricted our data analysis to voxels from an axial slice (*z* = 6 mm) covering the superior temporal plane.

## 3. Results

### 3.1. Repetition suppression

We report results on an exponential “item-lag” model, in which neuronal responses were modeled using Equation (2), *k* indexes the four stimulus types (HC, CT, RIN, Noise), and *r_k_* encodes the number of item repeats since the first stimulus of that type in the epoch. We also fitted “time-lag” models which used the same equation but where *r_k_* encoded the elapsed time (in seconds) since the first stimulus of that type in the epoch.

We first report a model comparison of the item-lag versus time lag models. Both model types were fitted to data from five sessions in six brain regions, giving a total of 30 data sets. The log model evidence was computed using a free energy approximation described earlier. The difference in log model evidence was then used to compute a log Bayes factor, with a value of 3 or greater indicating strong evidence.

Strong evidence in favor of the “time-lag” model was found in none out of 30 data sets, strong evidence in favor of the “item-lag” model was found in 22 out of 30 data sets. In the remaining 8 data sets, the Bayes factors were not decisive but the item-lag model was preferred in 7 of them. We therefore conclude that item-lags better capture the patterns in our data, and what follows below refers only to the item-lag models.

We now present results on the parametric responses of interest as captured by the β_*k*_ (initial response) and *a_k_* (decay) variables. These are estimated separately for each session of data using the model fitting algorithm described earlier. We then combine estimates over sessions using precision weighted averaging (Kasess et al., 2010). This is a Bayes-optimal procedure in which the overall parameter estimate is given by a weighted average of individual session estimates. The weights are given by the relative precisions (inverse variances) of the session estimates so that those with higher precision contribute more to the final parameter estimate.

**Figure 3:**
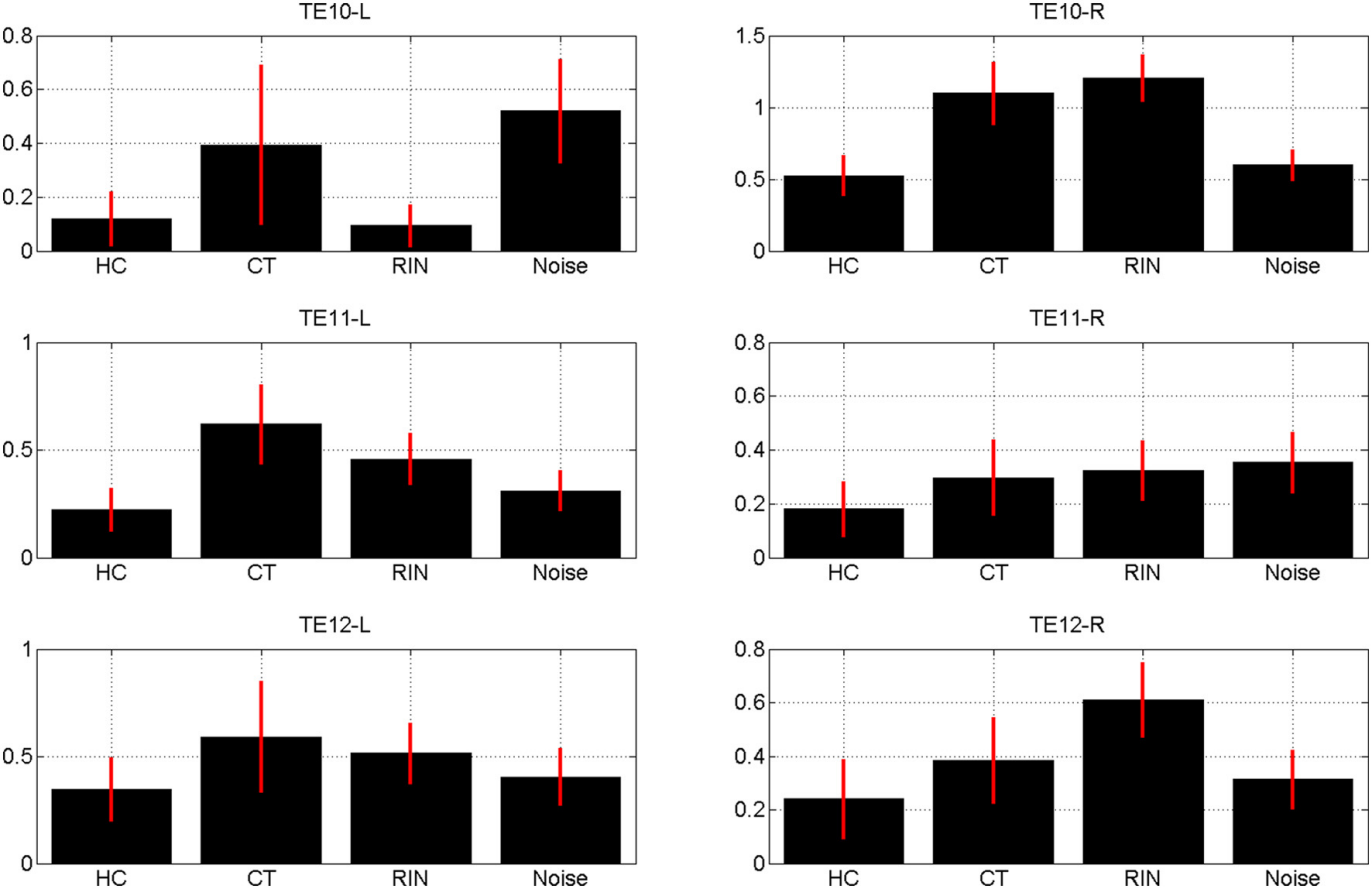
**Magnitude of initial response, β_*k*_ averaged over sessions, for conditions *k* = 1, 2, 3, 4 (Harmonic Complex—HC, Click Train—CT, Random Interval Noise—RIN, Noise)**. The red error bars indicate the standard deviations. Estimates are shown for the six regions of interest; TE10, TE11, and TE12 indicate primary, medial, and lateral regions of Heschl's Gyrus, and -L/-R indicates left/right hemisphere.

**Figure 4:**
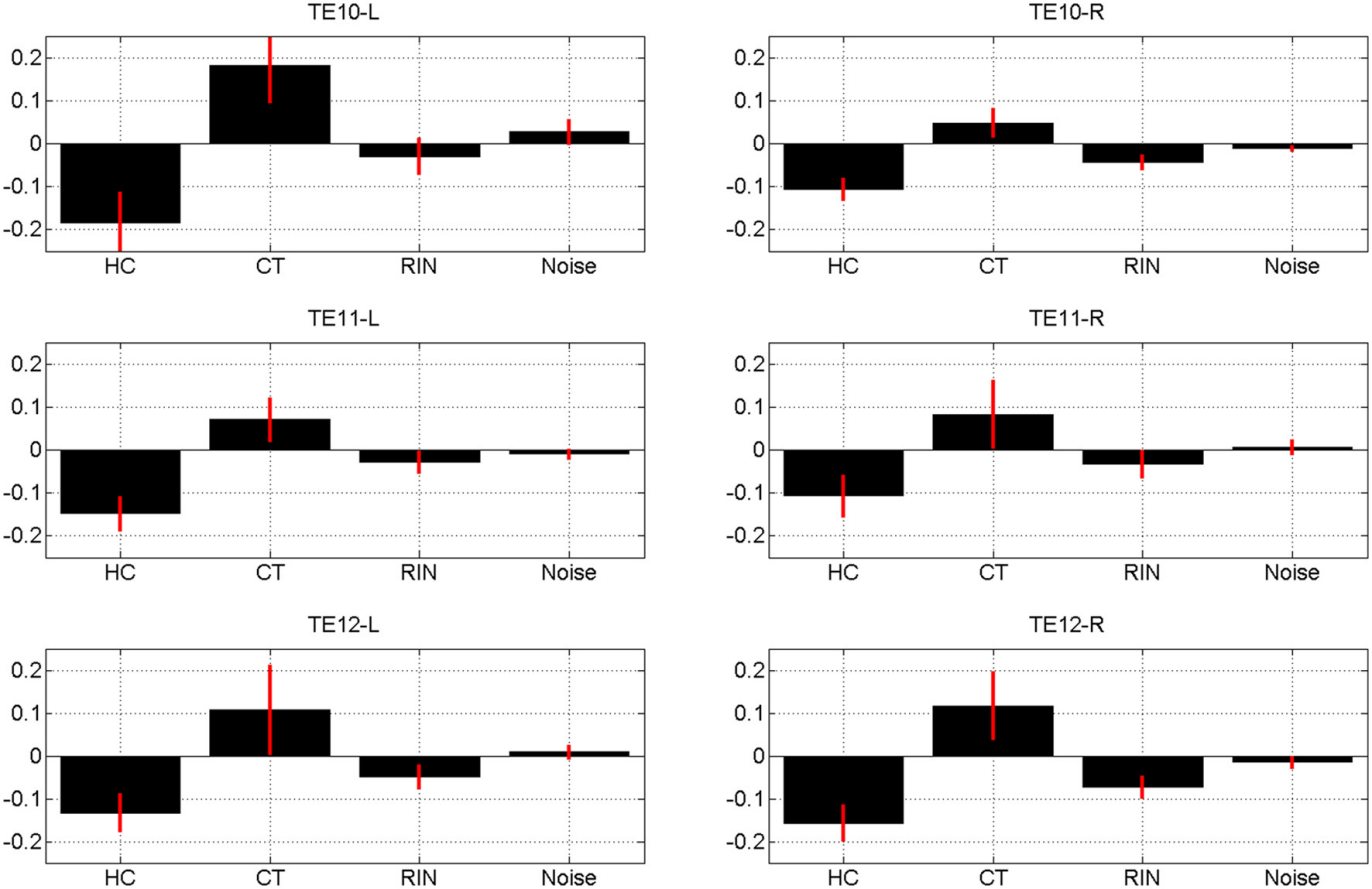
**Magnitude of repetition suppression effect, *a_k_* averaged over sessions, for conditions *k* = 1, 2, 3, 4 (Harmonic Complex—HC, Click Train—CT, Random Interval Noise—RIN, Noise)**. The red error bars indicate the standard deviations. Positive values indicate suppression and negative values facilitation. Estimates are shown for the six regions of interest; TE10, TE11, and TE12 indicate primary, medial, and lateral regions of Heschl's Gyrus, and -L/-R indicates left/right hemisphere.

The estimates of the initial response magnitudes, β_*k*_, are shown in Figure 3 and the estimates of the suppression effects, *a_k_*, are shown in Figure 4. Figure 3 shows that the pattern of initial responses (responses at item lag 0) is similar over all regions with CT and RIN typically eliciting the largest responses. Figure 4 shows that the noise stimulus does not elicit any repetition suppression effect in any region. The CT stimulus elicits a suppression effect which is strongest in TE10-L whereas the HC stimulus elicits a facilitation effect in all regions.

### 3.2. Tonotopic mapping

This section describes the estimation of Neural Response Functions for the Tonotopic Mapping data. We first focus on the Gaussian parametric form described in Equation (4). The Full Width at Half Maximum is given by $FWHM=2\sqrt{\left(2\ln2\right)}\sigma$. Following Moerel et al. (2012) we define the Tuning Value as *W* = μ/*FWHM* where μ and FWHM are expressed in Hz. Larger tuning values indicate more narrowly tuned response functions.

We restricted our data analysis to a single axial slice (z = 6) covering superior temporal plane. This slice contained 444 voxels in the auditory cortex.

**Figure 5:**
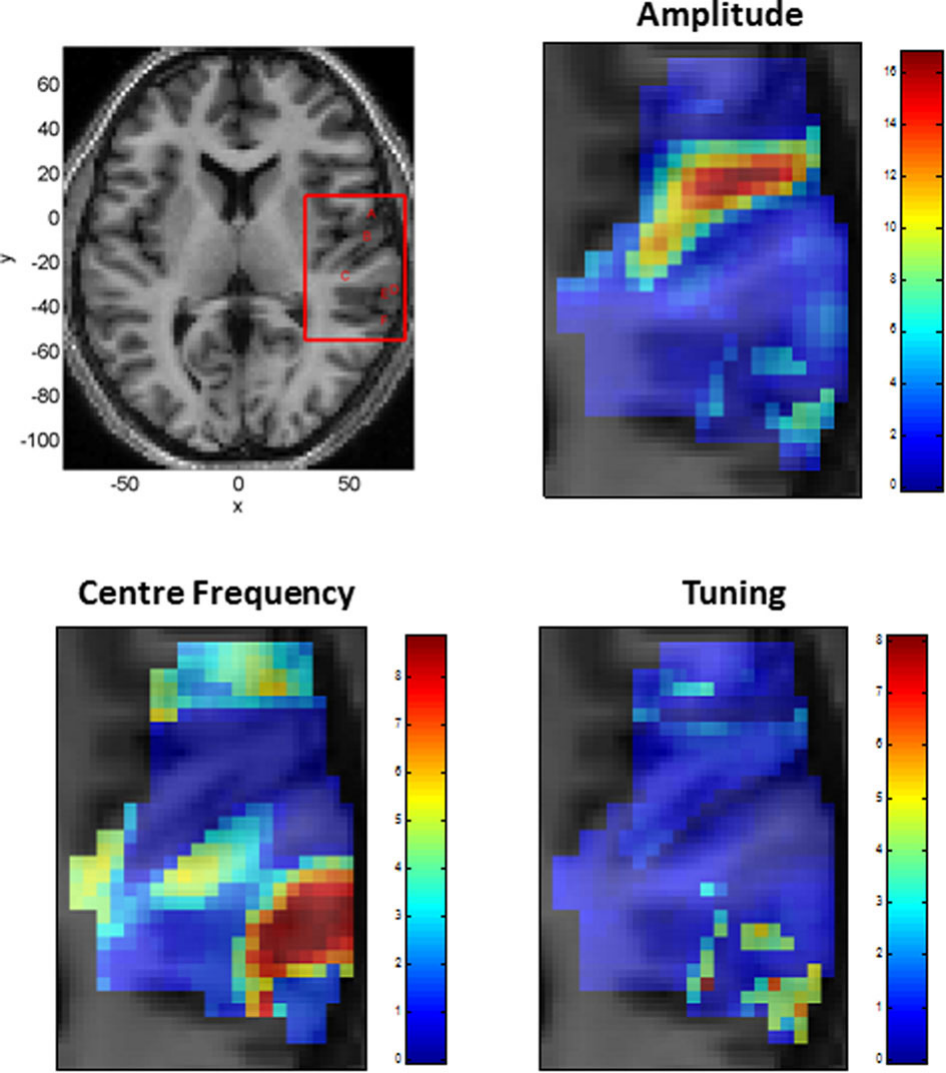
**Tonotopic Mapping with Gaussian neural response functions. Top Left:** Axial slice of structural image at *z* = 6 with bounding box showing region displayed on other three panels (x, y, and z denote MNI coordinates in mm). The labeling (A to F) refers to plots in Figures 6, 8. **Top Right:** Amplitude β (arbitrary units), **Bottom Left:** Center Frequency μ (kHz). **Bottom Right:** Tuning, *W* (ratio of Center Frequency to FWHM).

**Figure 6:**
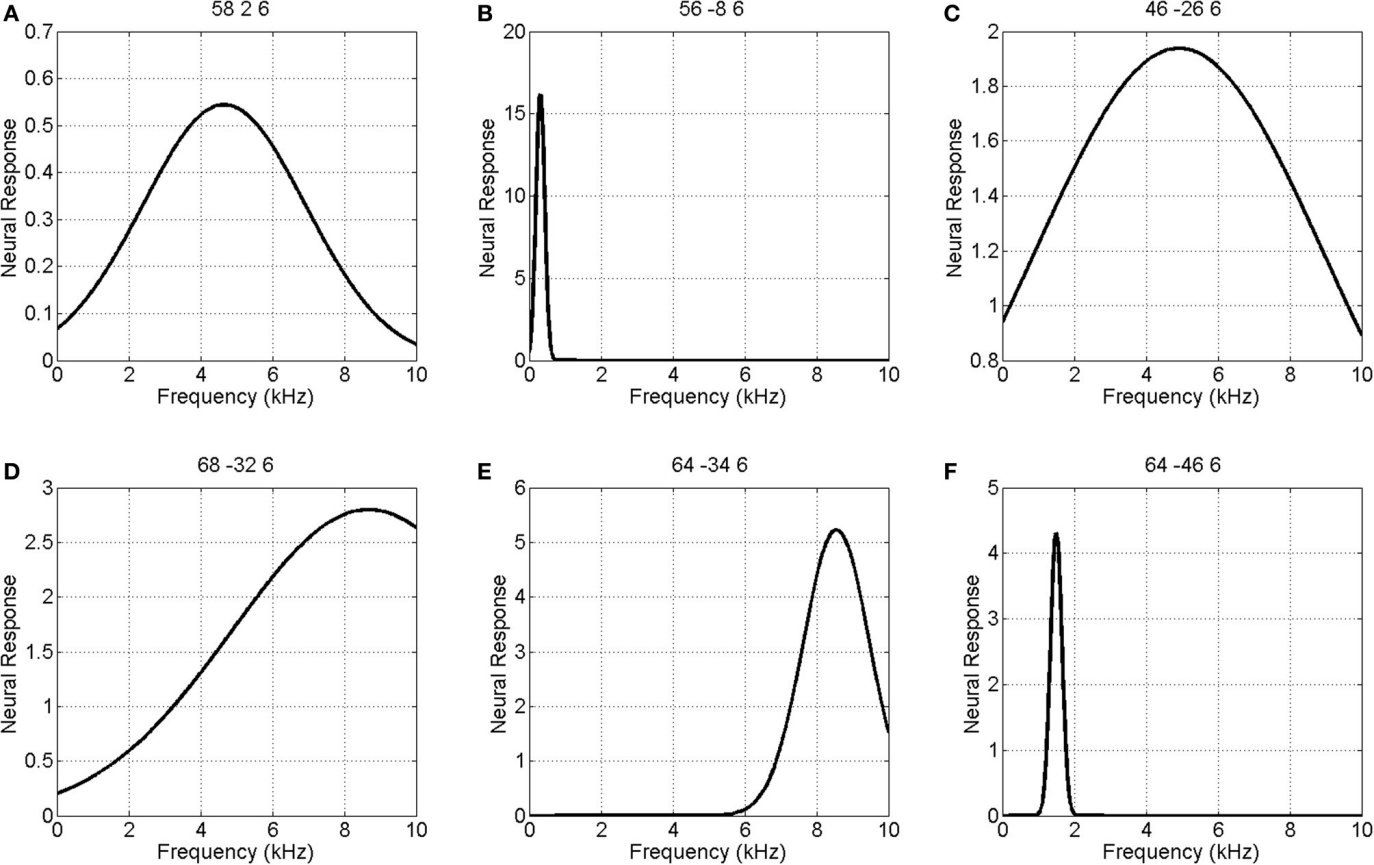
**Estimated Gaussian neural response functions at six selected voxels (voxel indices denote MNI coordinates in mm)**. The labeling (A – F) refers to positions shown in the top left panel of Figure 5.

Figure 5 shows the parameters of a Gaussian NRF as estimated over this slice. The main characteristics are as follows. First, the center frequency decreases and then increases again as one moves along the posterior to anterior axis with high frequencies at *y* = −30, low frequencies at *y* = −10 and higher frequencies again at *y* = 5. There is a single region of high amplitude responses that follows the length of Heschl's Gyrus (the diagonal band in the top right panel of Figure 5). These responses have a low center frequency of between 200 and 300 Hz. Finally the tuning values are approximately constant over the whole slice, with a value of about *W* = 1, except for a lateral posterior region with a much higher value of about *W* = 4. Figure 6 plots the estimated Gaussian response functions at six selected voxels.

**Figure 7:**
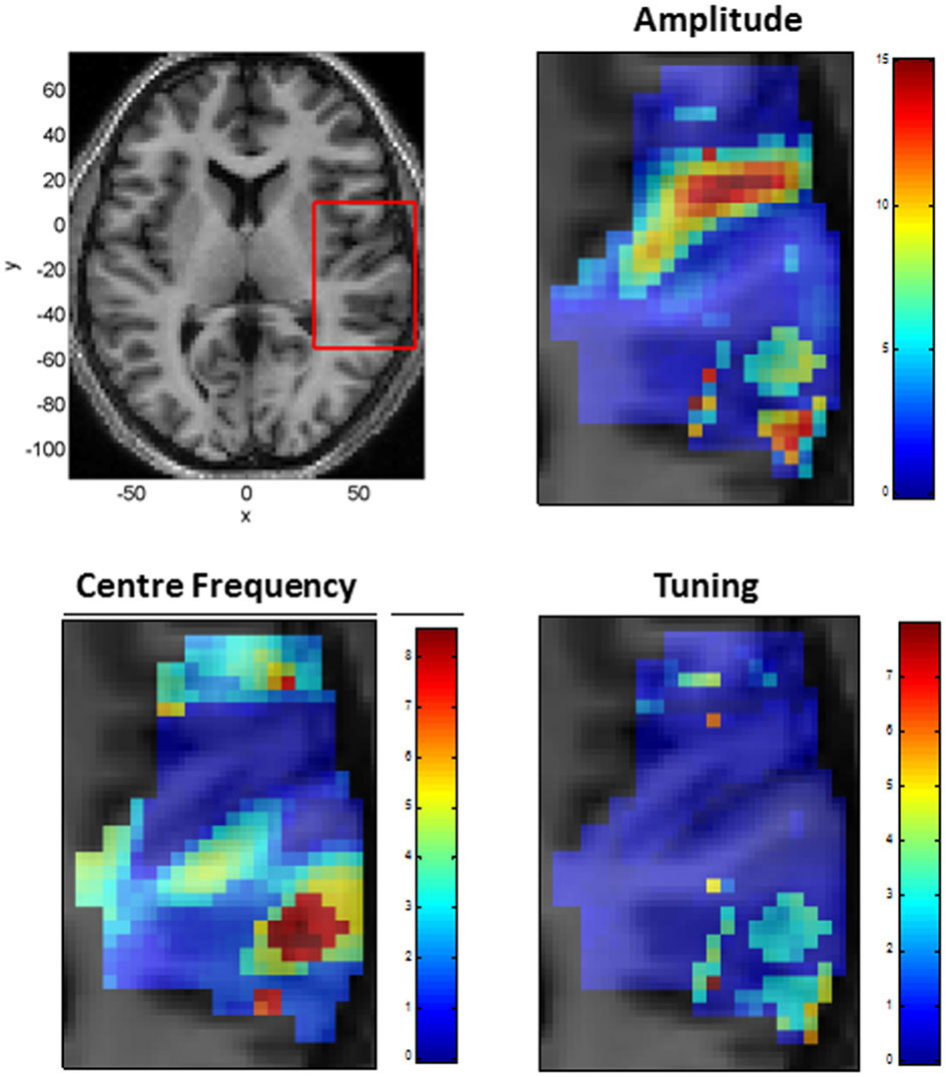
**Tonotopic Mapping with Mexican-Hat neural response functions. Top Left:** Axial slice of structural image at *z* = 6 with bounding box showing region displayed on other three panels (x, y, and z denote MNI coordinates in mm), **Top Right:** Amplitude β (arbitrary units). **Bottom Left:** Center Frequency μ (kHz). **Bottom Right:** Tuning, *W* (ratio of Center Frequency to FWHM).

**Figure 8:**
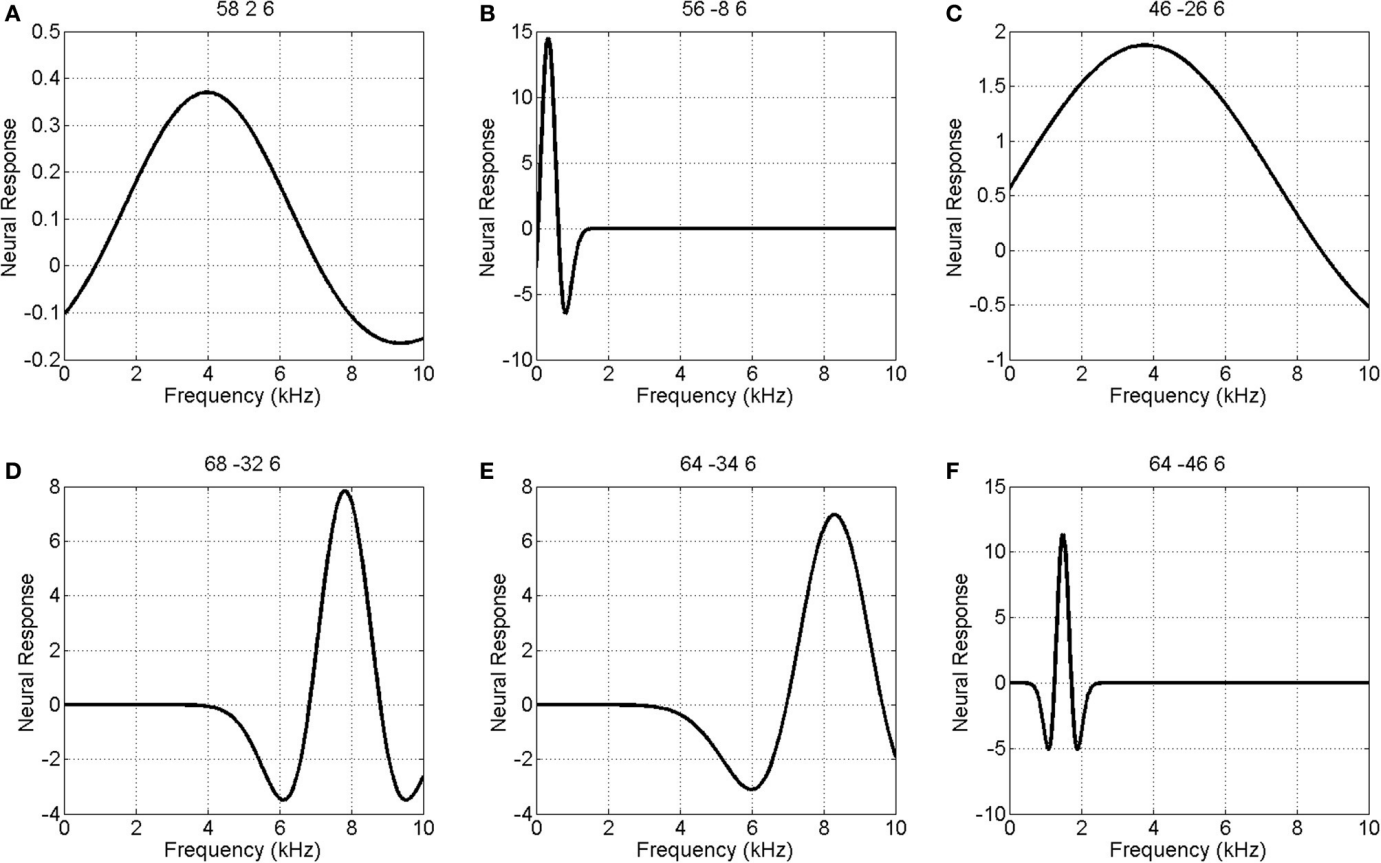
**Estimated Mexican-Hat neural response functions at six selected voxels (same voxels as in Figure 6)**. The labeling (A – F) refers to positions shown in the top left panel of Figure 5.

**Figure 9:**
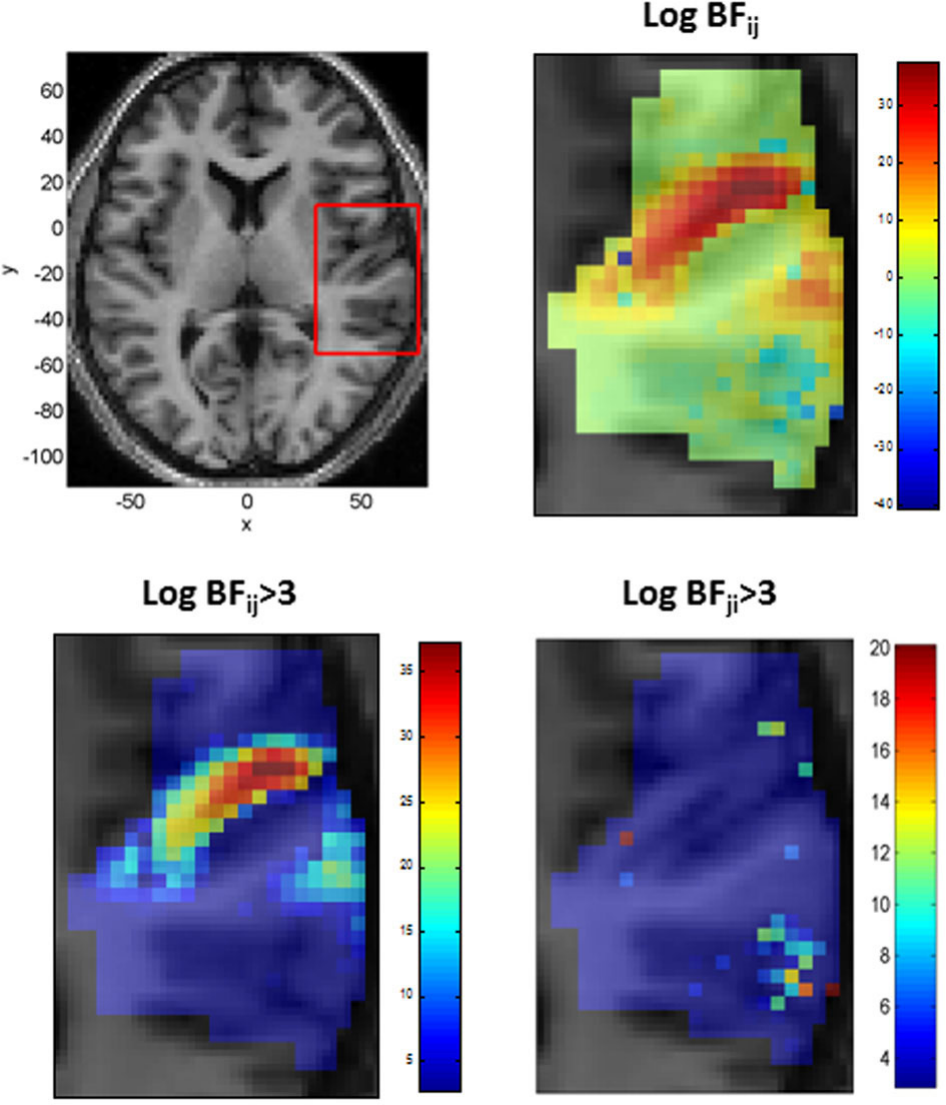
**Top Left:** Axial slice of structural image at *z* = 6 with bounding box showing region displayed on other three panels (x, y, and z denote MNI coordinates in mm), **Top Right:** Log Bayes factor for Gaussian versus Mexican-Hat NRFs (full range of values). Positive values provide evidence for the Gaussian and negative values for the Mexican-Hat NRF. **Bottom Left:** As top right but scale changed to range log *BF_ij_* > 3. **Bottom Right:** A plot of log *BF_ji_* over range log *BF_ji_* > 3 (i.e., in favor of Mexican-Hat). The Mexican-Hat is favored in a posterior region, and the Gaussian more anteriorly.

We also modeled neural responses using a Mexican-Hat wavelet function. Figure 7 plots the parameters of this NRF over the same slice through auditory cortex. The parameter estimates are very similar to those for the Gaussian NRFs, with minor differences in the lateral posterior region. Figure 8 shows the estimated NRFs for the same selected voxels as before, with the characteristic side lobes of the Mexican-Hat function clearly evident. Figure 9 plots a map of the log Bayes factor (see section 2.6) with positive values providing evidence for the Gaussian NRF and negative values providing evidence for the Mexican-Hat NRF. There was strong evidence (log *BF* > 3) in favor of the Gaussian model at 38% voxels and of the Mexican-Hat model (log *BF* < −3) at 8% voxels. Neither model is favored at the remaining 54% voxels. Figure 9 shows that the Mexican-Hat parametric form is favored in a posterior region, and the Gaussian function in more anterior regions.

### 3.3. Neuronal offsets

As the neuronal offset β_0_ (see Equations 3–5) is not part of the original HDM approach we were interested to see if the estimated offsets were significantly non-zero. This was assessed by computing the Bayes Factor in favor of a model with versus without such a term. This was computed using a Savage–Dickey approach which uses the prior and posterior distributions over parameters of the fitted model (and so does not require re-fitting of a model without the offset parameter) (Penny and Ridgway, 2013).

If the offset parameter is found to be useful for even a single fMRI time series then it is worth including in the model. Whilst it is true that adding parameters that don't explain variability in the data are deleterious for the model, we are in the fortunate situation of having many degrees of freedom to play with. This is because our models have the order of tens of parameters, whereas the fMRI time series comprise hundreds of time points.

For the Repetition Suppression data strong evidence (log *BF_ij_* > 3) in favor of models with an offset term was found for 6 out of 30 time series. For the Tonotopic Mapping data modeled with Gaussian NRFs strong evidence for the offset term was found for 192 out of 444 time series. For the Mexican-Hat NRF, strong evidence was found in 173 out of 444. We therefore conclude that it is useful to incorporate offset terms.

## 4. Discussion

This paper has proposed a methodology for estimating neural response functions from fMRI data. The method is based on a two-stage model comprising an NRF and an HRF that are together fitted to fMRI data using a Bayesian optimization algorithm that is widely used in neuroimaging (Friston et al., 2007). This algorithm has the added benefit of producing a model evidence score which we have used to provide a formal model comparison method (Penny, 2012) for evaluating alternative NRFs.

The work in this paper may be considered an advance from three different perspectives. The first views this work as an extension of the HDM, which uses the Balloon model to characterize the HRF, but constrains the relation between stimuli and neuronal activity to be purely categorical. The extension in this paper allows that relation to be parametric.

The second perspective views this work as an extension of linear models with “Parametric Modulation” terms, in which experimental variables of interest are used to modulate the height or duration of boxcar functions representing neuronal activity (Buchel et al., 1998; Grinband et al., 2008). The parametric modulators can reflect stimulus characteristics or, more generally, any experimental variable of interest. One use of the method is in “model-based fMRI” or “computational fMRI” in which computational models are first fitted to subjects behavioral data (reaction times and error rates) and the internal variables of these models are used as parametric modulators (O'Doherty et al., 2007; Friston and Dolan, 2009). The work in this paper represents an extension of this approach by allowing for non-linear relations between fMRI signals and unknown parametric variables. Whilst it is true that non-linear relationships can be accommodated in the linear framework by using a Taylor series approach, this has a number of disadvantages, as described in section 2.7.

The third perspective views this work as a novel method for the estimation of Population Receptive Fields (PRFs). These are similar, in principle, to receptive field functions derived for individual neurons from single unit electrophysiology (Dayan and Abbott, 2001) but estimate population rather than single neuron responses (Dumoulin and Wandell, 2008). In these studies parametric forms are derived for how neural responses depend on properties of sensory stimuli, such as orientation and contrast, in addition to spatial and temporal characteristics (Heckman et al., 2007; Dumoulin and Wandell, 2008; Kay et al., 2008).

Previously, a two-step procedure has been proposed for NRF estimation (Dumoulin and Wandell, 2008). The first step estimates an HRF assuming known neural activity, and the second estimates an NRF based on the estimated HRF. In this procedure the first step neglects the uncertainty in the assumed neural response and the second step neglects the uncertainty in the estimated HRF. This can lead to over-confident inferences. The simultaneous optimization of NRF and HRF parameters proposed in this paper, however, does not neglect these uncertainties. The conditional dependencies are captured in the relevant off-diagonal terms in the posterior covariance matrix and this guides parameter estimates during the optimization process (see Equations 27, 28). Additionally, models with highly correlated parameters also have lower model evidence (Penny, 2012), so this is also reflected in model comparison.

In this paper, we applied our method to investigate repetition suppression in the auditory system. Our model comparisons showed an exponential NRF based on item-lag was superior to one based on time-lag. Intuitively, one might think that if the brain charges and discharges some dynamical system then the time-lag model would be more likely than the item-lag model. However, it is well known that there are multiple brain systems for supporting discrete representations, as indicated for example by studies of numerosity (Dehaene and Brannon, 2011). Recent work in the visual domain has even characterized PRFs for numerosity (Harvey et al., 2013). Moreover, a dominant paradigm in the repetition suppression literature has assumed an item-lag like model, in which the number of repetitions is the key variable (Grill-Spector et al., 2006; Weigelt et al., 2008). This paper provides a methodology for testing such assumptions.

We found evidence of repetition suppression for the Click Train (CT) stimulus, facilitation for the Harmonic Complex (HC) in all the areas, facilitation for RIN in some areas (e.g., TE12R) and no suppression or facilitation for the noise stimulus. In our experiment the click trains (of a given pitch value) were identical within a trial, whereas the acoustic structure of HC, RIN and Noise varied within a trial (because of randomization of phase in the HC and use of a new exemplar of noise both for generation of RIN and Noise). The identical acoustic structure of CT and variation in acoustic structure in HC, RIN and Noise within trials may explain suppression of neural activity for CT and lack of it for HC, RIN and Noise.

We also applied our method to estimate tonotopic maps using two different functions: Gaussian and Mexican-Hat. The two functions produced maps which were similar. The results showed that low frequencies activated HG whereas regions posterior to HG were activated by high frequencies. This is in agreement with the tonotopic organization shown in previous works (Formisano et al., 2003; Talavage et al., 2004; Moerel et al., 2012). Bayesian comparison of the two models using Gaussian and Mexican-Hat functions showed that the former was preferred along the HG whereas the latter was the preferred model in regions posterior to HG. This is in agreement with a previous study (Moerel et al., 2013) that showed spectral profiles with a single peak in the central part of HG and Mexican-Hat like spectral profiles lying posterior to HG. We also observed broad tuning curves along the HG and narrow tuning curves posterior to HG. However, we did not observe the degree of variation in tuning width in areas surrounding HG, as was found in Moerel et al. (2012). This may be due to the fact that computations in our work were confined to a single slice. Further empirical validation is needed to produce maps of the tuning width covering wider areas of the auditory cortex.

A disadvantage of our proposed method is the amount of computation time required. For our auditory fMRI data (comprising 300 or 500 time points), optimization takes approximately 5 min per voxel/region on a desktop PC (Windows Vista, 3.2 GHz CPU, 12G RAM). One possible use of our approach could therefore be to provide “ballpark” estimates of NRF parameters, using data from selected voxels, and then to derive estimates at neighboring voxels using the standard Taylor series approach. Alternatively, optimization with a computer cluster should deliver results overnight for large regions of the brain (e.g., comprising thousands of voxels).

Our proposed method is suitable for modeling neural responses as simple parametric forms as assumed in previous studies using parametric modulators or population receptive fields. It could also be extended to simple non-linear dynamical systems, for example of the sort embodied in non-linear DCMs (Marreiros et al., 2008).

Two disadvantages of our approach are that there is no explicit model of ongoing activity, and it is not possible to model stochastic neural responses. Additionally, as the NRFs are identified solely from fMRI data our neural response estimates will not capture the full dynamical range of neural activity available from other modalities such as Local Field Potentials. On a more positive note, however, our approach does inherit two key benefits of fMRI; that it is a non-invasive method with a large field of view.

An additional finding of this paper is that model fits were significantly improved by including a neuronal offset parameter. This offset could also be included in Dynamic Causal Models (Friston et al., 2003) by adding an extra term to the equation governing vasodilation (Equation 7).

## Funding

William Penny is supported by a core grant [number 091593/Z/10/Z] from the Wellcome Trust: www.wellcome.ac.uk.

### Conflict of interest statement

The authors declare that the research was conducted in the absence of any commercial or financial relationships that could be construed as a potential conflict of interest.
